# Two sides of the same coin? Absolute income and perceived income inadequacy as social determinants of health

**DOI:** 10.1186/s12939-023-01945-z

**Published:** 2023-07-05

**Authors:** Rachelle Meisters, Polina Putrik, Daan Westra, Hans Bosma, Dirk Ruwaard, Maria Jansen

**Affiliations:** 1 Department of Health Services Research, Care and Public Health Research Institute (CAPHRI), Faculty of Health, Medicine and Life Sciences (FHML), Maastricht University, Duboisdomein 30, Maastricht, 6229 GT the Netherlands; 2 GGD Zuid Limburg, Academic Collaborative Centre for Public Health Limburg, Heerlen, the Netherlands; 3 Department of Social Medicine, Care and Public Health Research Institute (CAPHRI), Faculty of Health, Medicine and Life Sciences (FHML), Maastricht University, Maastricht, the Netherlands

**Keywords:** Health inequalities, Absolute income, Perceived income inadequacy, Mastery

## Abstract

**Background:**

Absolute income is commonly used in studies of health inequalities, however it does not reflect spending patterns, debts, or expectations. These aspects are reflected in measures concerning perceived income inadequacy. While health inequities by absolute income or perceived income inadequacy are well established, few studies have explored the interplay of absolute income and perceived income inadequacy in relation to health.

**Methods:**

Multiple data sources were linked into a nationally representative dataset (*n* = 445,748) of Dutch adults (18 +). The association between absolute income, perceived income inadequacy and health (self-reported health, chronic disease and psychological distress) was tested using logistic and Poisson regressions, controlling for various potential confounders (demographics, education) and mastery. Interactions were tested to check the association between perceived income inadequacy and health for different absolute income groups.

**Results:**

Perceived income inadequacy was reported at every absolute income group (with 42% of individuals in the lowest income group and 5% of individuals in the highest income group). Both absolute income and perceived income inadequacy were independently associated with health. The adjusted relative risk (RR) for lowest absolute income group is 1.11 (1.08–1.1.14) and 1.28 (1.24–1.32) for chronic disease and self-reported health respectively, and the Odds Ratio (OR) for psychological distress is 1.28 (1.16–1.42). For perceived income inadequacy the RR’s were 1.41 (1.37–1.46) and 1.49 (1.44–1.54) and the OR for psychological distress is 3.14 (2.81–3.51). Mastery appeared to be an important mediator for the relationship between perceived income inadequacy, poor self-rated health and psychological distress.

**Conclusions:**

Absolute income and perceived income inadequacy reflect conceptually different aspects of income and are independently associated with health outcomes. Perceived income inadequacy may be accounted for in health inequality studies, alongside measures of absolute income. In policy-making, targeting perceived income inadequacy might have potential to reduce health inequalities.

**Supplementary Information:**

The online version contains supplementary material available at 10.1186/s12939-023-01945-z.

## Introduction

Decades of research have established income as an important determinant of health [1–6]. In analyzing socioeconomic health inequalities, most studies account for an absolute measure of income [7, 8] from either questionnaires that inquire about annual or monthly household income levels [9], or tax-based registries [10]. Studies show that people with lower absolute incomes are limited in their resources to maintain good health [1], more likely to engage in unhealthy behaviors [11] and are more likely to experience psychological problems like anxiety and depression [4, 5, 9]. However, absolute income measures do not account for, for example, consumption, expense patterns, debts, aspirations or access to other economic resources [12].

Income inadequacy, on the other hand, does reflect spending patterns, expectations and unmet financial obligations such as debts, taking care of loved ones with chronic illnesses or disabilities, or financial obligations to family members. Having insufficient financial resources can affect health because people cannot afford healthy food or healthcare. In addition, perceived income inadequacy also relates to several psychological processes and can be influenced by social class, cultural and personality factors [8]. Research on perceived income inadequacy shows that it can, in turn, affect one’s functioning and health in a number of ways [7, 8, 13–17]. Regardless of their absolute income level, people who experience income inadequacy are not only worried about their financial constraints, the scarcity mindset theory poses that they are also impeded in their cognitive resources by constantly juggling, or being distracted by, expenses and trade-offs [13, 14]. These processes leave less cognitive resources available for other choices and actions in general and in health behaviors. Moreover, according to the risk sensitivity theory, perceived scarcity leads to increased risk taking behaviors [14]. In facing scarcities, individuals are more likely to make high-risk/high-reward decisions in order to get the necessary resources they require to satisfy their perceived (unmet) needs. Perceived income inadequacy is therefore not only about meeting actual financial obligations that are necessary for improving or keeping similar levels of health, but also about psychological processes hindering health. It is apparent from previous literature that absolute income and perceived income inadequacy reflect different aspects of income. People with lower absolute incomes do not always report income inadequacy [8] and individuals with comparable absolute incomes can report different levels of income inadequacy [12].

While both absolute income and perceived income inadequacy are important in studying health inequities, they are likely to impact health differently [7, 8]. In analyzing mental health in older adults, absolute financial measures had little effect, whilst perceived income inadequacy was found to be a predictor for anxiety and depression [16]. To complicate matters, mastery was found to (partially) explain the associations between low absolute income, perceived income inadequacy and poor mental health [18]. Mastery reflects the extent to which an individual believes the course of their live as being under their own control. People with low levels of mastery are considered more fatalistic, they believe their lives are set predeterminantly and they have no control over them [19]**.** Mastery seems to mediate the relation between income and health. Low socioeconomic status and perceived income inadequacy are associated with less mastery skills [20], and people with less mastery are more likely to experience poor mental health [21].

Whereas health inequities by absolute income or perceived income inadequacy are well established, few studies have explored the interplay of absolute income and perceived income inadequacy in relation to health. This study aims to 1) estimate the prevalence of income inadequacy across different absolute income levels, 2) investigate the association between perceived income inadequacy and health across different absolute income levels and 3) assess the role of demographic and socioeconomic confounders and mastery in these associations.

## Methods

This is a cross-sectional study of associations between absolute income, perceived income inadequacy, and health in the Netherlands for the year 2016. The data for this study were obtained from a combined dataset from the Dutch Public Health Survey and Statistics Netherlands. The Dutch Health Survey is administered once every four years by the Dutch Public Health Service, Statistics Netherlands, and the Dutch National Institute for Public Health and Environment (RIVM) to monitor local public health issues of the adult population. The Health Survey is completed either online, by paper and pencil, via telephone interviews or face-to-face, with a response rate of 40% in 2016 [22]. Survey weights were calculated to account for both the survey design (e.g. oversampling in some neighborhoods) and selective non-response (difference in response rate by age, sex, migration background and urbanization). The Health Survey data were enriched with data from Statistics Netherlands, based on the Personal Records database (migration background) and the Dutch Tax and Customs Administration Data (annual household income). The datasets were linked in the secured Statistics Netherlands environment via pseudonymized personal security codes. The linked dataset has been used in international publications before, for example, in analyzing the associations of loneliness in healthcare costs [23] and in socio-economic health inequalities [24].

### Measures

#### Outcome measures

Three dependent variables were used for this study to operationalize different aspects of health, namely ‘having at least one chronic disease’, ‘self-rated health’, and ‘psychological distress’. The operationalizations and sources of variables are listed in the Additional file 1: Table S1. In line with the European Statistics of Income and Living Conditions Survey (EU-SILC), the dichotomous variable ‘having at least one chronic disease’, was based on the question “Do you have one or more long-term disease (expected duration 6 months or longer)”. This question has been used in previous European studies as a proxy for health status [25–27]. Self-rated health was based on the question “In general, would you say your health is …”. Answer categories were given on a five-point Likert scale and dichotomized into “excellent or (very) good” or “fair or poor” health. The chronic disease question does not differentiate between physical or mental illnesses. To make sure mental health was also explicitly studied in relation to income, pshycological distress was included as an extra indicator of mental health. Psychological distress was measured with the Kessler (K10) psychological distress scale [28]. The K10 scale resulted in a score between 10 and 50. Based on national guidelines on the categorization of the K10, it was dichotomized into “none, low or moderate risk” (scores between 10 and 29) or “high risk” (scores between 30 and 50) for psychological distress [29]. For the K10 questionnaire, see Additional file 1: Table S2.

#### Income measures

In line with previous studies [7, 30], perceived income inadequacy was based on the question “In the past 12 months, have you had any concerns making ends meet with your household income?”. The answer categories included “No, no concerns”, “No, minor concerns”, “Yes, some concerns” or “Yes, major concerns”. Absolute income was based on the household income, as taken from the Statistics Netherlands registry. The household income represents all disposable income from labor and social benefits minus taxes and insurance premiums. The household income was standardized for the number of household members and then divided into quartiles based on the income distribution of the entire Dutch population. For the entirety of Dutch households, and therefore for this study sample, these quartiles represent the same monetary values. The 25^th^ percentile is equal to €18,200 annual standardized household income, the median is set at €25,200 and the 75^th^ percentile at €34,100.

#### Confounders

In line with previous research on the relationship between income inequality and mental health [4], the models are adjusted for age, sex, marital status (married, single, widowed or divorced), migration background (Dutch-born, Western migration background, non-western migration background) and highest level of completed education. The highest level of completed education was categorized into primary education, lower vocational education, secondary or middle vocational education, and higher vocational education or university degree. Mode of survey completion (internet, paper-and-pencil, face-to-face and telephone) was included as a control variable to rule out discrepancies due to the setting in which respondents were questioned. Mastery was based on the score for the seven-item Pearlin Mastery Scale [19]. Each item (see Additional file 1: Table S3 for the list of items) reflected an aspect of coping and answers were given on a five-point Likert scale (from totally disagree to totally agree), resulting in a score between 7 and 35.

### Statistical analyses

The relative risks (RR’s) for adverse health outcomes were modelled in a series of logistic and robust Poisson regressions. Since Odds Ratios (OR’s) estimated by logistic regressions do not appropriately approximate RR’s for so-called common outcomes (more than 10% of cases) [31], the outcomes ‘having at least one chronic disease’ and ‘self-rated health’ were modelled in Poisson regressions with robust variance. For the outcome ‘psychological distress’, the adverse outcome was present in approximately 5% of cases, and was therefore modelled in logistic regressions. For each outcome variable, a model was adjusted for age, sex, marital status, migration background, highest completed level of education, mastery, absolute household income and perceived income inadequacy. Next, interactions were tested between perceived income inadequacy and absolute income to check whether the association between perceived income inadequacy and health was different for different levels of absolute income. For significant interaction effects, stratified models were run. Given the fact that sociodemographic factors like age, sex, marital status, and migration status can be considered non-modifiable determinants of health and mastery a modifiable determinant, the added value of controlling for mastery was shown separately. Therefore, in the stratified models, and extra step was added in order to see the difference between adjustment with and without mastery for each absolute income group. All models were adjusted for mode of survey completion and accounted for complex survey design through survey weights. Missing data were imputed by means of Multiple Imputation by Chained Equations, (MICE, 5 imputations, *n* = 445,748) [32]. The significance level was set at alpha = 5%. Analyses were performed in Stata 16 [33].

## Results

### Descriptive statistics

Databases were linked for 445,748 individuals. The sample’s mean (SD) age was 59.4 (16.9) years and 54.2% of its respondents were female (Table 1). For migration background, 87.3% of the respondents were Dutch-born, 8.6% had a western migration background and 4.1% a non-western migration background. Most respondents were married or lived together (70.9%), 10.4% were single, 6.9% widowed, and 11.7% divorced. The majority of respondents reported adequate incomes, with 51.5% no concerns and 35.1% minor concerns. The other 13.5% reported inadequate incomes, with some concerns for 10.5% of respondents and 3.0% of respondents had major concerns. Primary school was the highest completed level of education for 7.5% of respondents, lower vocational education represented 33.5% of the sample, middle vocational or secondary education 30.4% and higher vocational or university degree represented 28.6% of the sample. The lowest household income quartile represented 14.6% of the sample, 27.5% of the respondents belonged to the second, 28.1% to the third and 29.8% to the highest income quartile. The mean (SD) score for mastery was 26.7 (5.2) (Table 1). Table 2 states the percentages of the different income inadequacy categories per standardized household income quartile. Perceived income inadequacy was present in all absolute income levels, even in the highest quartile, however at a much smaller scale (5%) than in the lowest income quartile (42%). The amount of missing responses per variable are presented in Table S4 of the Additional file 1.

**Table 1:** Sample characteristics (*n* = 445,748)

Sample Characteristics		N (%)
**Sex**	Male	204,095 (45.8%)
	Female	241,653 (54.2%)
**Migration background**	Dutch-born	389,298 (87.3%)
	Western background	38,445 (8.6%)
	Non-western background	18,005 (4,1%)
**Marital status**	Married/co-habitant	313,285 (70.9%)
	Single	45,853 (10.4%)
	Widowed	30,593 (6.9%)
	Divorced	51,877 (11.7%)
**Education**	Primary school	30,981 (7.5%)
	Lower vocational	138,947 (33.5%)
	Middle vocational/ secondary	125,981 (30.4%)
	Higher vocational/ university	118,985 (28.6%)
**Absolute income quartile**	0–25% (< €18,200)	64,825 (14.6%)
	26–50% (€18.201—€25.200)	122,251 (27.5%)
	51–75% (€25.201—€34.100)	125,196 (28.1%)
	76–100% (> €34.100)	132,739 (29.8%)
**Perceived income inadequacy**	Major concerns	12,367 (3.0%)
	Some concerns	43,640 (10.5%)
	Minor concerns	146,380 (35.1%)
	No concerns	215,147 (51.5%)
**Chronic disease**	None	261,977 (59.9%)
	At least one	175,086 (40.1%)
**Self-rated health**	Fair, bad	125,043 (28.4%)
	(very) good, excellent	315,079 (71.6%)
**Psychological distress**	No or low risk	411,536 (95.1%)
	High risk	21,362 (4.9%)
**Mode of survey completion**	Paper	221,433 (49.7%)
	Internet	223,657 (50.2%)
	Face-to-face	428 (0.1%)
	Telephone	230 (0.01%)
		**Mean (sd)**
**Age**		59.4 (16.9)
**Mastery**		26.7 (5.2)

Self-reported variables: marital status, education, perceived income inadequacy, chronic disease, self-rated health, psychological distress and mastery. Registry data variables: age, sex, migration background, absolute income quartile

**Table 2:** Percentage of perceived income inadequacy category per absolute income quartile

		**Perceived income inadequacy**			
		No concerns	Minor concerns	Some concerns	Major concerns
**Absolute income quartile**	0–25%	19%	38%	27%	15%
	26–50%	33%	44%	18%	5%
	51–75%	50%	38%	10%	2%
	76%-100%	71%	24%	4%	1%

Based on weighted data after multiple imputation

### Absolute income and perceived income inadequacy

Table 3 shows that after correcting for age, sex, marital status, migration background, highest completed level of education and mastery, low absolute income and perceived income inadequacy are both independently associated with poorer health across all three outcome variables. The associations were strongest for perceived income inadequacy and psychological distress. For the lowest absolute income quartile, the RR’s are 1.11 (1.08–1.14) and 1.28 (1.24–1.32) for chronic disease and self-rated respectively, and the OR for psychological distress is 1.28 (1.16–1.42). For major income inadequacy concerns, the RR’s for chronic disease and self-rated health are 1.41 (1.37–1.46) and 1.49 (1.44–1.54) and the OR for psychological distress is 3.14 (2.81–3.51; Table 3).

**Table 3:** Associations between absolute income, perceived income inadequacy and health outcomes (*n* = 445,748)

RR/OR (95% CI)		**Chronic disease**	**Poor self-rated health**	**Psychological distress**
**Absolute income quartile**	0–25%	**1.11 (1.08–1.14)**	**1.28 (1.24–1.32)**	**1.28 (1.16–1.42)**
	26–50%	**1.04 (1.02–1.06)**	**1.19 (1.16–1.22)**	**1.13 (1.03–1.25)**
	51–75%	1.01 (0.99–1.03)	**1.10 (1.07–1.13)**	1.10 (0.99–1.21)
	76%-100%	ref	ref	ref
**Perceived income inadequacy**	Major concerns	**1.41 (1.37–1.46)**	**1.49 (1.44–1.54)**	**3.14 (2.81–3.51)**
	Some concerns	**1.32 (1.29–1.35)**	**1.49 (1.45–1.53)**	**2.03 (1.86–2.21)**
	Minor concerns	**1.15 (1.14–1.17)**	**1.24 (1.22–1.27)**	**1.41 (1.30–1.52)**
	No concerns	ref	ref	ref

All models include age, sex, marital status, migration background, highest completed level of education, mastery, absolute income quartile and perceived income inadequacy. Analyses are based on weighted, multiple-imputed data. Associations in bold are significant *p* < 0.05

**Fig. 1:**
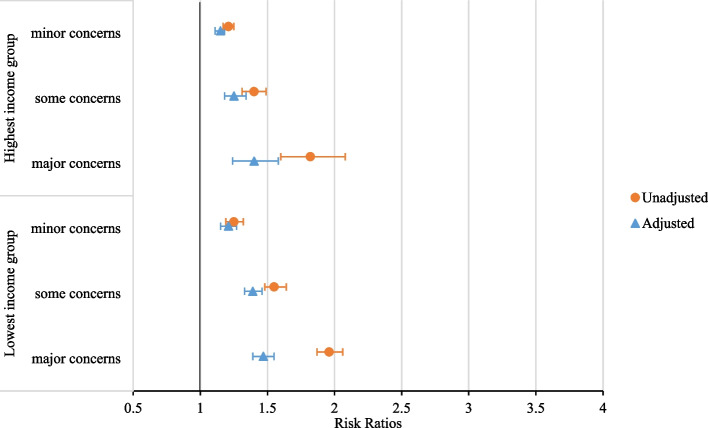
Associations of perceived income inadequacy with chronic disease, for highest and lowest income quartile in unadjusted and fully adjusted models. Lowest income group: 0%-25% household income percentile, highest income group: 75%-100% household income percentile. Unadjusted models only include absolute income and perceived income inadequacy. The adjusted model includes age, sex, marital status, migration background, highest completed level of education, mastery, absolute income quartile and perceived income inadequacy. Analyses are based on weighted, multiple-imputed data

**Fig. 2:**
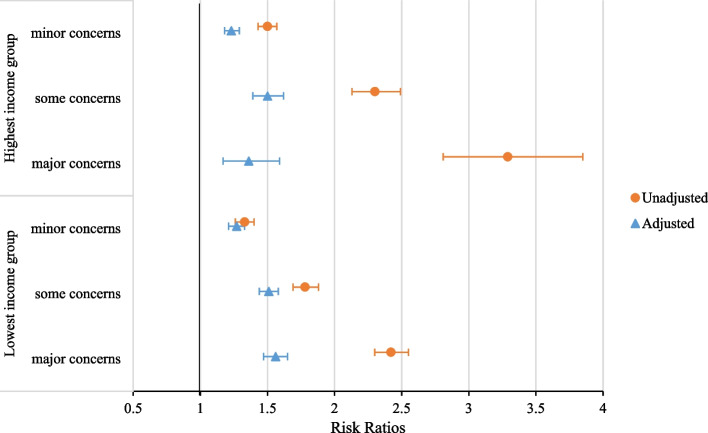
Associations of perceived income inadequacy with self-rated health, for highest and lowest income quartile in unadjusted and fully adjusted models. Lowest income group: 0%-25% household income percentile, highest income group: 75%-100% household income percentile. Unadjusted models only include absolute income and perceived income inadequacy. The adjusted model includes age, sex, marital status, migration background, highest completed level of education, mastery, absolute income quartile and perceived income inadequacy. Analyses are based on weighted, multiple-imputed data

The interaction tests between absolute income and perceived income inadequacy were significant for all three health outcomes, however, stratified analyses revealed no relevant patterns. The adjusted and unadjusted results for highest and lowest income group are presented in Fig. 1 (chronic disease), Fig. 2 (self-rated health) and for Additional file 1: Figure S1 (psychological distress). In unadjusted models, the effect sizes of perceived income inadequacy were higher for self-rated health and mental health in higher absolute income groups (Fig. 2, Additional file 1: Figure S1 and Table S5)***.*** In fully adjusted models, the effect sizes of income inadequacy leveled out across absolute income groups (Figs. 1 and 2, Additional file 1: Figure S1 and Table S5). Further analyses showed that of all confounders, mastery was mainly responsible for the decrease of effect sizes in all income groups, the attenuation is slightly higher for higher income groups (Additional file 1: Table S5). As a sensitivity analysis, the associations between income inadequacy were stratified for age groups. For chronic disease and self-rated health, the interactions between income inadequacy and age group was significant. For both outcomes, stratified results are presented in the Additional file 1: Table S6. The RR’s for both outcomes were the highest for the youngest age group (19–40 years old) and the lowest for the oldest age group (81 years and older).

## Discussion and conclusion

This study estimated the prevalence of perceived income inadequacy across absolute income levels and investigated the associations between perceived income inadequacy and health across absolute income levels in a nationally representative sample of the Dutch adult population. First, the results show that perceived income inadequacy concerns are reported at every level of absolute income: from 5% of the people in the highest absolute income group to 42% of individuals in the lowest income group. This indicates that these measures address conceptually different aspects of income as even the highest income group members report income inadequacy. This suggests that focusing on either absolute income or perceived income inadequacy is likely not sufficient in studies of health inequalities. Second, the results show that both absolute income and perceived income inadequacy are independently associated with health. Of all three health outcomes used in this study, perceived income inadequacy was most strongly related to poorer mental health. Third, this study compared the association of perceived income inadequacy and health across different income groups and found similar patterns in the highest and lowest absolute income groups. In other words, independent from the income one earns, facing income inadequacies has similar negative associations with their health, after adjusting for socio-demographic confounders and mastery. These similar patterns were found after including sociodemographic factors and mastery in the models. Before inclusion of mastery, perceived income inadequacy was more strongly associated with poor self-rated health and psychological distress. This suggests that mastery may have a mediating role in perceived income inadequacies and health as consistent with previous research [18]. Our results suggest that mastery can help explain the impact of perceived income inadequacy on health.

The findings are consistent with previous research in finding perceived income inadequacy to occur in all income groups [8], however being more prevalent in the lowest income group. In line with other studies, associations were found between perceived income inadequacy and poorer self-rated health [7, 8, 34] and poorer mental health [16]. Moreover, these studies also controlled for absolute income measures and found the associations of perceived income inadequacy on health to be independent of absolute income. Arber, Fenn and Meadows found in their UK sample that in later life, perceived income inadequacy is associated with poorer self-rated health and absolute income was no longer associated with health [7]. Arber et al. suggest that their results support the argument that measures of perceived income inadequacy may be an ‘even a better predictor of self-rated health than income’ [7, 35]. Similar findings were presented in an Italian-based study [36]. Cialani and Mortazavi found absolute income levels to be of lesser importance to health once adjusting for perceived income inadequacy. This difference could be potentially attributed to the measurement of absolute income. The current study used registry data for absolute income and the study by Cialani and Mortazavi used self-reported measures. Respondents may find reporting their net annual household income correctly too complicated, or too privacy-sensitive. As Cialani and Mortazavi indicated, there was a lack of sufficient detail in the absolute income indicator (annual income) [36]. Another possibility is that the observed difference is context specific and the studied associations work differently across countries or cultures. Further (cross-national) research is needed to investigate this.

For public health policymakers and researchers, the findings suggest that perceived income inadequacy could be another potential determinant of health in addition to absolute income, especially for mental health. Further research with suitable designs is needed to understand the complexity and directions of the relationships between income and health. Perceived income concerns can arise at any income level and its relation to poorer health is similar in all absolute income groups. This finding points policymakers at a potential target for other interventions to reduce health inequities, in addition to policies aimed at raising absolute incomes at the low end. More qualitative research is needed to unravel causes of perceived income inadequacy at different absolute income levels. The insights gained from qualitative research could be informative in developing additional policies and interventions to tackle perceived income inadequacy. For example, by simplifying financial rules and arrangements, by helping citizens in understanding and using these arrangements or by providing citizens with support in balancing income and spending patterns. A few existing examples include a peer-to-peer intervention based in a primary care setting in Canada [37]. Guided by a trained facilitator, groups of participants (in a similar stage of life) helped each other in improving their understanding of personal finances, taxes, benefits, savings and practice skills such as budgeting, collaboration, decision making and problem solving. After completing the intervention, the majority of participants reported a higher optimism towards their financial situation, a higher degree of financial control and lower finance-related stress [37]. Positive results have also been found in the UK when implementing Citizen Advice Bureaus in general practitioner settings for both users and providers [38].

This study is not without limitations. First, as this study uses cross-sectional data, no causal conclusions can be drawn. We cannot conclude that absolute income and perceived income inadequacy lead to poorer health or conversely, that poor health results into lower absolute incomes and in turn, in perceived income inadequacy. Second, a selection bias may be present in the sample as it is known that people with low SES and/or poor health are less likely to participate in survey research [39]. The Public Health Service and Statistics Netherlands have taken this underrepresentation into account in their survey design by oversampling low SES groups and by providing weighted data for their dataset. Despite oversampling and weighted data, the reported associations of perceived income inadequacy and health may still represent conservative estimates. Third, the personal trait of negative affect may influence responses in terms of self-rated health and perceived income inadequacy [40]. Those respondents who are more likely to experience negative emotions may have completed their evaluation of health and income inadequacy both more negatively. Future research with longitudinal design is warranted to draw causal conclusions, preferably together with possibilities to control for negative affectivity in surveys, for example with the Positive and Negative Affect scales (PANAS) [41].

## Conclusions

Perceived income inadequacy is present in all income groups, with even the highest income earners reporting inadequate incomes. Our findings indicate that perceived income inadequacy could be a potential determinant of health in addition to absolute income, especially for mental health. As such, perceived income inadequacy may be accounted for in health inequality studies in addition to absolute measures of income.

## Supplementary Information

**Additional file 1: Table S1.** Categories, operationalization, and sources of dependent and independent variables. **Table S2.** Questions for psychological distress (Kessler 2002) [K10]^1^. **Table S3.** Statements for mastery (Pearlin and Schooler 1978)^2^. **Table S4.** Missing data (*n*=445.748). **Table S5.** Associations of perceived income inadequacy with health outcomes, per absolute income quartile in unadjusted and fully adjusted models. **Figure S1.** Associations of perceived income inadequacy with pshycological distress, for highest and lowest income quartile in unadjusted and adjusted models. **Table S6.** Associations of income inadequacy with health outcomes, per age group.

## Data Availability

The dataset was provided by Statistics Netherlands and the Dutch Public Health Services. Requests to access these datasets should be directed to Statistics Netherlands, microdata@cbs.nl. Results are based on calculations by researchers from Maastricht University using non-public microdata from Statistics Netherlands.
