# Supplementary material for: Two sides of the same coin? Absolute income and perceived income inadequacy as social determinants of health
Source: Int J Equity Health. 2023 Jul 5;22:128. doi: 10.1186/s12939-023-01945-z (PMC10324279; doi:10.1186/s12939-023-01945-z)
Supplement: Supplementary file 1 — Additional file 1: Table S1. Categories, operationalization, and sources of dependent and independent variables. Table S2. Questions for psychological distress (Kessler 2002) [K10]1. Table S3. Statements for mastery (Pearlin and Schooler 1978)2. Table S4. Missing data (n=445.748). Table S5. Associations of perceived income inadequacy with health outcomes, per absolute income quartile in unadjusted and fully adjusted models. Figure S1. Associations of perceived income inadequacy with pshycological distress, for highest and lowest income quartile in unadjusted and adjusted models. Table S6. Associations of income inadequacy with health outcomes, per age group. [file 12939_2023_1945_MOESM1_ESM.docx]

**Additional file 1**

| Table S1. Categories, operationalization, and sources of dependent and independent variables. | | | |
| --- | --- | --- | --- |
| Variable | **Category** | **Coded** | **Source** |
| Age^§,1^ | 19-40 | 0 | Statistics Netherlands |
|  | 41-64 | 1 | Statistics Netherlands |
|  | 65-80 | 2 | Statistics Netherlands |
|  | 81+ | 3 | Statistics Netherlands |
| Sex^§^ | male | 0 | Statistics Netherlands |
|  | female | 1 | Statistics Netherlands |
| Migration status^§^ | Dutch born | 0 | Statistics Netherlands |
|  | Western migration background | 1 | Statistics Netherlands |
|  | Non-western migration background | 2 | Statistics Netherlands |
| Marital status* | Married or living together | 0 | Health survey |
|  | Single | 1 | Health survey |
|  | Divorced | 2 | Health survey |
|  | Widowed | 3 | Health survey |
| Education* | Primary school | 3 | Health survey |
|  | Lower vocational | 2 | Health survey |
|  | Middle vocational/ secondary | 1 | Health survey |
|  | Higher vocational/ university | 0 | Health survey |
| Household income  quartile^§^ | 0-25% | 3 | Statistics Netherlands |
|  | 26-50% | 2 | Statistics Netherlands |
|  | 51-75% | 1 | Statistics Netherlands |
|  | 76-100% | 0 | Statistics Netherlands |
| Perceived income inadequacy* | Major concerns | 3 | Health survey |
|  | Some concerns | 2 | Health survey |
|  | Minor concerns | 1 | Health survey |
|  | No concerns | 0 | Health survey |
| Mastery*^,a^ | Score 7-35 | n/a | Health survey |
| Mode of survey completion | Paper | 0 | Health survey |
|  | Internet | 1 | Health survey |
|  | Face-to-face interview | 2 | Health survey |
|  | Telephone interview | 3 | Health survey |
| Chronic disease* | None | 0 | Health survey |
|  | At least one | 1 | Health survey |
| Self-rated health* | (Very) good, excellent | 0 | Health survey |
|  | Fair, bad | 1 | Health survey |
| Psychological distress*^,b^ | No or low risk (score 10-29) | 0 | Health survey |
|  | High risk (score 30-50) | 1 | Health survey |

§Registry variables. *Self-reported variables. ¹Age is used as a continuous variable in the regression models and used as a categorical variable in analyzing the interaction effects between age groups and income inadequacy. ^a^Mastery is a continuous variable. The seven statements (see Table A3) are answered on a 5-point Likert scale, leading to a score between 7 and 35. A score of 19 or lower is considered as insufficient mastery. ^b^Psychological distress is based on the Kessler-10 questionnaire. The 10 questions (see Table A2) are answered on 5-point Likert scale, leading to a score between 10 (no risk) and a maximum of 50 (high risk).

| Table S2. Questions for psychological distress (Kessler 2002) [K10]^1^. |
| --- |
| 1. About how often did you feel tired for no good reason? |
| 1. About how often did you feel nervous? |
| 1. About how often did you feel so nervous that nothing could calm you? |
| 1. About how often did you feel hopeless? |
| 1. About how often did you feel restless of fidgety? |
| 1. About how often did you feel so restless that you could not sit still? |
| 1. About how often did you feel depressed? |
| 1. About how often did you feel that everything was an effort? |
| 1. About how often did you feel so sad that nothing could cheer you up? |
| 1. About how often did you feel worthless? |

^1^The K10 questions are answered on a 5-point Likert scale (always, usually, sometimes, occasionally, or never).

| Table S3. Statements for mastery (Pearlin and Schooler 1978)^2^. |
| --- |
| 1. I have little control over the things that happen to me. |
| 1. There is really no way that I can solve some of the problems I have. |
| 1. There is little I can do to change many of the important things in my life. |
| 1. I often feel helpless in dealing with the problems of life. |
| 1. Sometimes I feel that I am being pushed around in life. |
| 1. What happens to me in the future mostly depends on me. |
| 1. I can do just about anything I really set my mind to do. |

^2^ The 7 statements by Pearlin & Schooler are answered on a 5-point Likert scale (totally agree, agree, neither agree or disagree, disagree or totally disagree).

| **Table S4. Missing data (n=445.748)** | |
| --- | --- |
| Variable | N(%) |
| Age^§^ | 0 (0%) |
| Sex^§^ | 0 (0%) |
| Migration background^§^ | 0 (0%) |
| Marital status* | 4,140 (0.9%) |
| Education* | 30,854 (6.9%) |
| Absolute income quartile^§^ | 737 (0.2%) |
| Perceived income inadequacy* | 2,814 (6.3%) |
| Mastery* | 29,196 (6.5%) |
| Chronic disease* | 8,685 (1.9%) |
| Self-rated health* | 5,626 (1.3%) |
| Psychological distress* | 12,850 (2.9%) |
| Mode of survey completion^§^ | 0 (0%) |

^§^ Registry variables, *self-reported variables.

| Table S5. Associations of perceived income inadequacy with health outcomes, per absolute income quartile in unadjusted and fully adjusted models. | | | | | | | |
| --- | --- | --- | --- | --- | --- | --- | --- |
| RR/OR  (95% CI) | **Absolute income quartile** | | | | | | |
|  | **0-25%** | | | | **26-50%** | | |
| Chronic Disease | Model 1. | | Model 2. | Model 3. | Model 1. | Model 2. | Model 3. |
| Major concerns | | **1.96 (1.87-2.06)** | **1.96 (1.86-2.06)** | **1.47 (1.39-1.55)** | **1.70 (1.61-1.80)** | **1.93 (1.82-2.04)** | **1.43 (1.35-1.51)** |
| Some concerns | | **1.55 (1.48-1.64)** | **1.60 (1.52-1.68)** | **1.39 (1.33-1.46)** | **1.39 (1.34-1.44)** | **1.53 (1.48-1.58)** | **1.33 (1.28-1.38)** |
| Minor concerns | | **1.25 (1.19-1.32)** | **1.28 (1.22-1.35)** | **1.21 (1.15-1.27)** | **1.20 (1.17-1.24)** | **1.23 (1.20-1.27)** | **1.16 (1.13-1.19)** |
| No concerns | ref | | ref | ref | ref | ref | ref |
| Self-rated health | | |  |  |  |  |  |
| Major concerns | | **2.42 (2.30-2.55)** | **2.36 (2.23-2.49)** | **1.56 (1.47-1.65)** | **2.39 (2.25-2.52)** | **2.64 (2.49-2.79)** | **1.59 (1.50-1.69)** |
| Some concerns | | **1.78 (1.69-1.88)** | **1.84 (1.76-1.94)** | **1.51 (1.44-1.58)** | **1.62 (1.55-1.68)** | **1.82 (1.74-1.89)** | **1.44 (1.38-1.49)** |
| Minor concerns | | **1.33 (1.26-1.40)** | **1.38 (1.32-1.45)** | **1.27 (1.21-1.33)** | **1.24 (1.20-1.28)** | **1.29 (1.25-1.33)** | **1.17 (1.14-1.21)** |
| No concerns | ref | | ref | ref | ref | ref | ref |
| Psychological distress | | | |  |  |  |  |
| Major concerns | | **9.56 (8.21-11.14)** | **8.25 (7.03-9.68)** | **3.02 (2.47-3.70)** | **12.86 (10.91-15.16)** | **10.82 (9.10-12.86)** | **3.31 (2.69-4.06)** |
| Some concerns | | **3.32 (2.86-3.86)** | **3.12 (2.68-3.63)** | **1.92 (1.60-2.31)** | **3.76 (3.32-4.27)** | **3.57 (3.14-4.07)** | **1.97 (1.70-2.29)** |
| Minor concerns | | **1.58 (1.34-1.87)** | **1.60 (1.36-1.90)** | **1.33 (1.07-1.65)** | **1.68 (1.49-1.89)** | **1.68 (1.49-1.90)** | **1.35 (1.18-1.54)** |
| No concerns | ref | | ref | ref | ref | ref | ref |

Model 1 is an unadjusted model, it only includes absolute income quartile and perceived income inadequacy. Model 2 (not tabulated here) is included in Table A6 of the appendix. Model 2 is adjusted for age, sex, marital status, migration background, highest completed level of education, absolute income quartile and perceived income inadequacy. Model 3 is a fully adjusted model and includes age, sex, marital status, migration background, highest completed level of education, mastery, absolute income quartile and perceived income inadequacy. Analyses are based on weighted, multiple-imputed data. Associations in bold are significant p<0.05.

| Table S5. Continued | | | | | | | |
| --- | --- | --- | --- | --- | --- | --- | --- |
| RR/OR  (95% CI) | **Absolute income quartile** | | | | | | |
|  | **51-75%** | | | **76-100%** | | | |
| Chronic Disease | Model 1. | Model 2. | Model 3. | Model 1. | Model 2. | Model 3. | |
| Major concerns | **1.82 (1.69-1.98)** | **1.95 (1.81-2.11)** | **1.37 (1.27-1.47)** | **1.82 (1.60-2.08)** | **1.97 (1.74-2.24)** | | **1.40 (1.24-1.58)** |
| Some concerns | **1.39 (1.33-1.45)** | **1.51 (1.45-1.58)** | **1.27 (1.22-1.33)** | **1.40 (1.31-1.49)** | **1.50 (1.41-1.60)** | | **1.25 (1.18-1.34)** |
| Minor concerns | **1.17 (1.14-1.21)** | **1.22 (1.19-1.26)** | **1.14 (1.11-1.17)** | **1.21 (1.17-1.25)** | **1.25 (1.21-1.29)** | | **1.15 (1.11-1.18)** |
| No concerns | ref | ref | ref | ref | ref | ref | |
| Self-rated health | | | | | | | |
| Major concerns | **3.04 (2.79-3.30)** | **2.96 (2.72-3.23)** | **1.47 (1.35-1.60)** | **3.29 (2.01-3.85)** | **3.00 (2.56-3.51)** | | **1.36 (1.17-1.59)** |
| Some concerns | **1.95 (1.85-2.06)** | **2.09(1.98-2.21)** | **1.49 (1.41-1.57)** | **2.30 (2.13-2.49)** | **2.27 (2.10-2.47)** | | **1.50 (1.39-1.62)** |
| Minor concerns | **1.38 (1.33-1.44)** | **1.44 (1.39-1.51)** | **1.26 (1.22-1.31)** | **1.50 (1.43-1.57)** | **1.48 (1.42-1.56)** | | **1.23 (1.18-1.29)** |
| No concerns | ref | ref | ref | ref | ref | ref | |
| Psychological distress | | | | | | | |
| Major concerns | **14.10 (11.45-17.36)** | **10.95 (8.78-13.65)** | **2.68 (2.01-3.58)** | **16.72 (12.40-22.55)** | **11.06 (8.08-15.14 )** | | **3.22 (2.12-4.90)** |
| Some concerns | **4.76 (4.10-5.53)** | **4.17 (3.57-4.88)** | **2.07 (1.73-2.49)** | **5.47 (4.49-6.66)** | **4.24 (3.44-5.24)** | | **2.08 (1.63-2.65)** |
| Minor concerns | **2.07 (1.80-2.37)** | **1.97 (1.72-2.26)** | **1.46 (1.25-1.71)** | **2.29 (1.99-2.64)** | **2.00 (1.72-2.32)** | | **1.42 (1.19-1.70)** |
| No concerns | ref | ref | ref | ref | ref | ref | |

Model 1 is an unadjusted model, it only includes absolute income quartile and perceived income inadequacy. Model 2 (not tabulated here) is included in Table A6 of the appendix. Model 2 is adjusted for age, sex, marital status, migration background, highest completed level of education, absolute income quartile and perceived income inadequacy. Model 3 is a fully adjusted model and includes age, sex, marital status, migration background, highest completed level of education, mastery, absolute income quartile and perceived income inadequacy. Analyses are based on weighted, multiple-imputed data. Associations in bold are significant p<0.05.

**Figure S1. Associations of perceived income inadequacy with psychological distress, for highest and lowest income quartile in unadjusted and adjusted models.**

Lowest income group: 0%-25% household income percentile, highest income group: 75%-100% household income percentile. Unadjusted models only include absolute income and perceived income inadequacy. Adjusted models includes age, sex, marital status, migration background, highest completed level of education, mastery, absolute income quartile and perceived income inadequacy. Analyses are based on weighted, multiple-imputed data.

| **Table S6. Associations of income inadequacy with health outcomes, per age group.** | | | | | |
| --- | --- | --- | --- | --- | --- |
| **Health outcome** |  | **Age group** | | | |
|  | RR (95% CI) | **19-40** | **41-64** | **65-80** | **81+** |
| **Chronic disease** | Inadequate, major concerns | **1.67 (1.54-1.81)** | **1.39 (1.34-1.44)** | **1.29 (1.25-1.32)** | **1.16 (1.08-1.25)** |
|  | Inadequate, some concerns | **1.53 (1.44-1.63)** | **1.31 (1.27-1.35)** | **1.30 (1.27-1.32)** | **1.18 (1.14-1.23)** |
|  | Adequate, minor concerns | **1.25 (1.19-1.32)** | **1.16 (1.14-1.19)** | **1.15 (1.13-1.16)** | **1.09 (1.06-1.11)** |
|  | Adequate, no concerns | ref | ref | ref | ref |
| **Self-rated health** | Inadequate, major concerns | **1.88 (1.70-2.06)** | **1.59 (1.52-1.66)** | **1.37 (1.32-1.42)** | **1.19 (1.12-1.26)** |
|  | Inadequate, some concerns | **1.82 (1.68-1.98)** | **1.65 (1.59-1.71)** | **1.42 (1.39-1.45)** | **1.22 (1.17-1.27)** |
|  | Adequate, minor concerns | **1.48 (1.38-1.58)** | **1.34 (1.29-1.38)** | **1.22 (1.20-1.24)** | **1.14 (1.12-1.17)** |
|  | Adequate, no concerns | ref | ref | ref | ref |

All models are adjusted for sex, marital status, migration background, highest completed level of education and self-mastery.
Analyses are based on weighted, multiple-imputed data. Associations in bold are significant p<0.05
